# Kinetic model-informed deep learning for multiplexed PET image separation

**DOI:** 10.1186/s40658-024-00660-0

**Published:** 2024-07-01

**Authors:** Bolin Pan, Paul K. Marsden, Andrew J. Reader

**Affiliations:** School of Biomedical Engineering and Imaging Sciences, King’s College London, London, UK

**Keywords:** Multiplexed PET, Kinetic modeling, Spectral analysis, Physics-informed deep learning

## Abstract

**Background:**

Multiplexed positron emission tomography (mPET) imaging can measure physiological and pathological information from different tracers simultaneously in a single scan. Separation of the multiplexed PET signals within a single PET scan is challenging due to the fact that each tracer gives rise to indistinguishable 511 keV photon pairs, and thus no unique energy information for differentiating the source of each photon pair.

**Methods:**

Recently, many applications of deep learning for mPET image separation have been concentrated on pure data-driven methods, e.g., training a neural network to separate mPET images into single-tracer dynamic/static images. These methods use over-parameterized networks with only a very weak inductive prior. In this work, we improve the inductive prior of the deep network by incorporating a general kinetic model based on spectral analysis. The model is incorporated, along with deep networks, into an unrolled image-space version of an iterative fully 4D PET reconstruction algorithm.

**Results:**

The performance of the proposed method was evaluated on a simulated brain image dataset for dual-tracer [$^{18}$F]FDG+[$^{11}$C]MET PET image separation. The results demonstrate that the proposed method can achieve separation performance comparable to that obtained with single-tracer imaging. In addition, the proposed method outperformed the model-based separation methods (the conventional voxel-wise multi-tracer compartment modeling method (v-MTCM) and the image-space dual-tracer version of the fully 4D PET image reconstruction algorithm (IS-F4D)), as well as a pure data-driven separation [using a convolutional encoder-decoder (CED)], with fewer training examples.

**Conclusions:**

This work proposes a kinetic model-informed unrolled deep learning method for mPET image separation. In simulation studies, the method proved able to outperform both the conventional v-MTCM method and a pure data-driven CED with less training data.

## Introduction

Positron emission tomography (PET) is a medical imaging technique that enables direct and quantitative observations of tissue radioactivity over time in vivo. The use of different PET radiotracers facilitates the measurement of various aspects of tumour metabolism for diagnosis, characterization, and monitoring of the response to therapy, as well as the measurement of neurotransmitter release and receptor densities in brain studies [1, 2]. Due to the radioactive and biological half-lives involved, it is usually not possible to perform more than one tracer acquisition in a single patient visit. To obtain information on, for example, glucose metabolism with [$^{18}$F]FDG (radioactive half-life=109.8 mins) and protein synthesis with [$^{11}$C]MET (radioactive half-life=20.4 mins), two scans may be conducted separately [3, 4] (scanning [$^{11}$C]MET first and then allowing a long time delay before scanning [$^{18}$F]FDG). This extends the scanning time for the patient and only works for comparable simple combinations of radiotracers. To fully exploit the range of tracers now available, it would be useful to scan more than one tracer simultaneously.

Multiplexed PET (mPET) imaging allows for the synchronization of observing physiological and pathological information from multiple tracers in a single scan, reducing the total examination time and providing complementary information for the characterization of disease. In mPET imaging, multiple tracers are injected with a time offset anywhere from zero to several minutes, and the dynamic/static imaging measurements of each individual tracer are then recovered in a single scan. However, the separation of the mPET signals within a single PET scan is challenging due to the fact that each tracer gives rise to indistinguishable 511 keV photon pairs, and thus no unique energy information for differentiating the source of each photon pair.

Research on mPET imaging has been ongoing for the past two decades. The separation of the mPET signals was initially proposed based on the significant differences in the radioactive decay of each tracer [5], which was further investigated by Verharghe et al. [6] and Figueiras et al. [7]. Another widely studied method is based on multi-tracer compartment modeling (MTCM) which was first proposed by Koeppe et al. [8] for estimating the kinetic parameters of $^{11}$C-labelled tracers. Ikoma et al. [9] then applied the MTCM method for dual-tracer [$^{18}$F]FDG+[$^{11}$C]flumazenil separation. The feasibility of the MTCM method in mPET imaging, exploring various dual-tracer combinations through the analysis of simulated PET data and preclinical PET data, were investigated in [1, 2, 10–14]. Black et al. further extended the MTCM method from dual-tracer imaging to triple-tracer imaging [15]. The MTCM method is highly sensitive to noise and is prone to fall into local minima, even when the noise level is low, due to the non-linearity of the fitting problem. In order to improve the separation performance of the MTCM method, Zhang et al. [16] proposed a reformulation of the conventional multi-tracer compartment model using fewer parameters by separating the linear part from the nonlinear part [16]. On this basis, the separable parameter space technique was incorporated with PET image reconstruction to reduce the influence of the noise in the fitting process for dual-tracer [$^{18}$F]FDG+[$^{11}$C]MET PET image separation [17]. However, the aforementioned MTCM-based methods assume that the arterial input function (AIF) of each tracer is known. This implies that, in practice, separating the measured (dual-tracer) AIFs is necessary prior to MTCM. Kudomi et al. [18] and Taheri et al. [19] introduced non-invasive model-based methods to separate dual-tracer AIFs. However, these methods are highly dependent on the shape of the curves. Thus, there remains a lack of an accurate non-invasive method for AIF separation. Verhaeghe and Reader [20] proposed using a general kinetic model based on spectral analysis [21] to separate [$^{18}$F]FDG and multiple [$^{15}$O] water injections without the need for any AIFs to be known. Despite its advantages of not requiring any AIFs for separation, the solution to the fitting problem in this method is non-unique.

Other model-based methods not limited to the compartment model have been investigated for mPET signal separation, such as principal component analysis [1], generalised factor analysis [22], the reference-region model [23] and basis pursuit [24]. However, these methods assume a long time-delayed injection protocol and that the AIF of each tracer is known. A machine learning-based method based on the recurrent extreme gradient boosting algorithm was proposed to separate both the dual-tracer AIF and the dual-tracer time-activity curves (TACs) in a region of interest (ROI), allowing a shorter delay between the injection of two tracers than that of the MTCM method [25]. The mPET signal can also be separated based on the case where an additional high-energy $\gamma$ photon is emitted along with a positron for one of the two tracers, and thus the different isotopes can be discriminated in the measured data [26–29]. However, these methods are only valid for some tracer combinations: a purely positron emitting isotope and a positron-$\gamma$ emitting isotope, e.g., $^{18}$F and $^{60}$Cu, limiting the selection of tracers.

The huge recent success of using deep learning (DL) for PET image processing and reconstruction [30, 31] has seen an increasing interest in applying similar strategies to mPET image separation. Many applications of DL for mPET image separation have been concentrated on pure data-driven methods, e.g., training a neural network on separating mPET images into single-tracer dynamic/static images from either reconstructed dynamic mPET images (post-separation) [32–38] or mPET sinogram sequences (direct separation) [39–41]. These methods use over-parameterized networks with only a very weak inductive prior and require large quantities of training data.

In this paper, we strengthen the inductive prior of the deep separation network by embedding a general kinetic model for dynamic dual-tracer [$^{18}$F]FDG+[$^{11}$C]MET PET imaging without explicitly knowing the AIFs, ensuring network outputs are consistent with known kinetic models. In particular, we incorporate a general kinetic model based on spectral analysis into a neural network by unrolling a regularised implementation of an image-space version of an iterative fully 4D PET image reconstruction algorithm [42].

## Methods

### Spectral analysis kinetic model

**Fig. 1:**
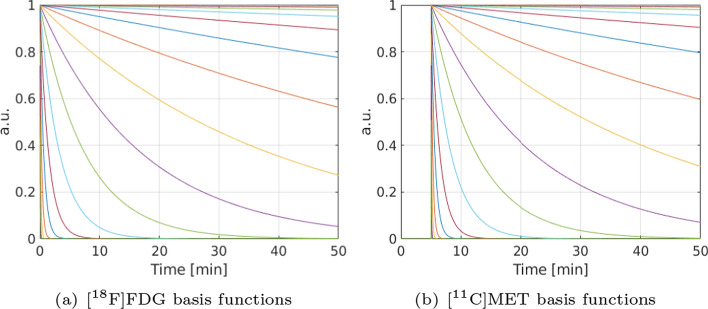
An example of a set of predetermined (finely sampled) decaying exponential temporal basis function for (a) [$^{18}$F]FDG and (b) [$^{11}$C]MET with a 5 mins time-delayed injection (by setting the early values equal to zero). For both FDG and MET, 25 exponential temporal basis function were chosen and the decay constants were logarithnmically spaced between 0.001 and 5 min$^{-1}$, including the special case of the decay constant equal to zero

The modeling of the tracer activity concentrations (i.e., the TACs) at a voxel or within an ROI in an image involves the convolution of the measured AIF with a model-dependent tissue unit impulse response, where the number of compartments is predefined. In our work, we proposed use of a more general kinetic model that makes no a priori assumption regarding to the number of compartments necessary to model the data. Based on spectral analysis [21], the single-tracer activity concentrations (i.e., single-tracer TACs) for all voxels in an image can be described using a linear model [20, 42]

1 $$\begin{aligned} {\textbf{f}} = \textbf{HBc}, \end{aligned}$$

where ${\textbf{c}} \in {\mathbb {R}}^{JN\times 1}_{\ge 0}$ contains the spectral coefficients for each of the *N* predetermined exponential temporal basis functions for all *J* voxels, ${\textbf{B}} \in {\mathbb {R}}^{JT\times JN}$ contains the *N* exponential temporal basis functions sampled at *T* time points (as a repeated $T\times N$ sub-matrix placed at diagonally consecutive positions along ${\textbf{B}}$, the contents of which are shown in Fig. 1(a) for FDG and (b) for MET, respectively), and ${\textbf{H}}\in {\mathbb {R}}^{JT\times JT}_{\ge 0}$ convolves each of the time functions resulting from $\textbf{Bc}$ with a global generating function ${\textbf{h}}\in {\mathbb {R}}^{T\times 1}_{\ge 0}$ (shared across all voxels). Note that model (1) allows for the shapes of the generating function ${\textbf{h}}$ to encompass an infinite number of possibilities, with the AIF being a special case of ${\textbf{h}}$. The linear model (1) can be extended to the dual-tracer ([$^{18}$F]FDG+[$^{11}$C]MET) TACs for all voxels

2 $${\textbf{f}}_{\rm{D}}= {\textbf{f}}_{\rm{FDG}} + {\textbf{f}}_{\rm{MET}}$$

3 $$= \begin{bmatrix} {\textbf{H}}_{\rm{FDG}} {\text{ }} {\textbf{H}}_{\rm{MET}} \end{bmatrix} \begin{bmatrix} {\textbf{B}}_{\rm{FDG}} &{} {\textbf{0}}\\ {\textbf{0}} &{} {\textbf{B}}_{\rm{MET}} \end{bmatrix} \begin{bmatrix} {\textbf{c}}_{\rm{FDG}}\\ {\textbf{c}}_{\rm{MET}} \end{bmatrix}$$

4 $$\begin{aligned}&= {\textbf{H}}_{\rm{D}}{\textbf{B}}_{\rm{D}}{\textbf{c}}_{\rm{D}} , \end{aligned}$$

where ${\textbf{B}}_{\rm{FDG}}\in {\mathbb{R}}^{JT\times JE}$ contains the *E* exponential temporal basis function for FDG, ${\textbf{B}}_{\rm{MET}}\in {\mathbb{R}}^{JT\times JF}$ contains the *F* exponential temporal basis functions for MET, ${\textbf{B}}_{\rm{D}}$ is a block diagonal matrix with ${\textbf{B}}_{\rm{FDG}}$ and ${\textbf{B}}_{\rm{MET}}$ on the diagonal, $${\mathbf{H}}_{\rm{D}} = \begin{bmatrix} {\textbf{H}}_{\rm{FDG}} {\text{ }} {\textbf{H}}_{\rm{MET}} \end{bmatrix}$$, and $${\mathbf{c}}_{\rm{D}} = \begin{bmatrix} {\textbf{c}}_{\rm{FDG}} {\text{ }}{\textbf{c}}_{\rm{MET}} \end{bmatrix}^T$$.

### Fully 4D inspired dual-tracer PET image separation

Integrating the linear model (4) for dual-tracer voxel-wise TACs with the fully 4D algorithm proposed for PET image reconstruction [20, 42] and considering regularisation on the coefficients ${\textbf{c}}_{\rm{D}}$, leads to finding a solution to the following variational minimization problem

5 $$\begin{aligned} ({\hat{{\textbf{c}}}_{\rm{D}}, {\hat{\textbf{h}}}_{\rm{D}}}) = \mathop {{{\,\mathrm{arg\,min}\,}}}\limits _{\textbf{h}_{\rm{D}}, \textbf{c}_{\rm{D}}\ge \textbf{0}} \rm{KL}({\textbf{m}}, \textbf{H}_{\rm{D}}\textbf{B}_{\rm{D}}\textbf{c}_{\rm{D}}) + \lambda {\mathcal {R}}({\textbf{c}}_{\rm{D}}), \end{aligned}$$

where ${\textbf{m}}\in {{\mathbb {R}}^{JT\times 1}}$ is the measured dual-tracer activity images (e.g., voxel-wise dual-tracer TACs), ${\mathcal {R}}: {\mathbb {R}}^{J(E+F)\times 1}\rightarrow {\mathbb {R}}$ is the regulariser and $\lambda >0$ is the regularisation hyperparameter. Note that in $${\mathbf{H}}_{\rm{D}} = \begin{bmatrix} {\textbf{H}}_{\rm{FDG}} {\text{ }} {\textbf{H}}_{\rm{MET}} \end{bmatrix}$$, matrices ${\textbf{H}}_{\rm{FDG}}$ and ${\textbf{H}}_{\rm{MET}}$ contain the tracer-specific global generating functions ${\textbf{h}}_{\rm{FDG}}$ and ${\textbf{h}}_{\rm{MET}}$, respectively. This means that $\mathbf{H}_{\rm{D}}$ depends on $$\mathbf{h}_{\rm{D}} = \begin{bmatrix} {\textbf{h}}_{\rm{FDG}} {\text{ }} {\textbf{h}}_{\rm{MET}} \end{bmatrix}$$, and thus, we denote $\mathbf{h}_{\rm{D}}$ as one of the optimization variables in (5). The minimization problem (5) can be solved by alternating minimization for each variable while keeping the other fixed as suggested in [20, 42], resulting in the following iterative scheme (index *k*)

$$\begin{aligned} \hat{{\textbf{c}}}_{\rm{D}}^{k+1}&= \mathop {{{\,\mathrm{arg\,min}\,}}}\limits _{\textbf{c}_{\rm{D}}\ge \textbf{0}} \mathrm {KL({\textbf{m}}}, {\textbf{H}}_{\rm{D}}^{k}\mathbf{B}_{\rm{D}}{\textbf{c}}_{\rm{D}}) + \lambda {\mathcal {R}}({\textbf{c}}_{\rm{D}})\qquad \qquad \qquad \quad ({\textbf {c-update}}) \end{aligned}$$

$$\begin{aligned} \hat{{\textbf{h}}}_{\rm{D}}^{k+1}&= \mathop {{{\,\mathrm{arg\,min}\,}}}\limits _{\textbf{h}_{\rm{D}}\ge \textbf{0}} \mathrm {KL({\textbf{m}}}, \mathbf{H}_{\rm{D}}\mathbf{B}_{\rm{D}}{\textbf{c}}_{\rm{D}}^{k+1}) .\qquad \qquad \qquad \quad ({\textbf {h-update}}) \end{aligned}$$

If ${\mathcal {R}}$ are closed proper convex, the **c-update** can be solved using the proximal gradient descent algorithm [43], resulting in the following iteration (index *i*)

6 $$\begin{aligned} {{\bar{\textbf{c}}}}^i_{\rm{D}}&= {\textbf{c}}_{\rm{D}}^i - \mu ^i \nabla \mathrm {KL}({\textbf{m}}, {\textbf{H}}_{\rm{D}}^k\mathbf{B}_{\rm{D}}{\textbf{c}}_{\rm{D}}^i) \end{aligned}$$

7 $$\begin{aligned} {\textbf{c}}_{\rm{D}}^{i+1}&= \mathop {{{\,\mathrm{arg\,min}\,}}}\limits _{\textbf{c}_{\rm{D}}\ge \textbf{0}} \frac{1}{2}||{\textbf{c}}_{\rm{D}} - {{\bar{\textbf{c}}}}^i_{\rm{D}}||_2^2 + \lambda \mu ^{i}{\mathcal {R}}({\textbf{c}}_{\rm{D}}) \end{aligned}$$

8 $$\begin{aligned}&= (\textbf{prox}_{\lambda \mu ^i {\mathcal {R}}}( {{\bar{\textbf c}}}^i_{\rm{D}}))_{+}, \end{aligned}$$

where $\mu ^{i} > 0$ is a step size in the *i*th iteration, $\textbf{prox}(\cdot )$ is the proximal operator w.r.t. ${\mathcal {R}}$, and the subscript “$+$” indicates the non-negativity constraint on the spectral coefficients ${\textbf{c}}_{\rm{D}}$. By choosing $\mu ^i = {\textbf{c}}_{\rm{D}}^i/({({\textbf{H}}_{\rm{D}}^k\mathbf{B}_{\rm{D}})^T {\textbf{1}}})$, the gradient descent step (6) can be simplified as an MLEM update [44]

9 $$\begin{aligned} \bar{{\textbf{c}}}^i_{\rm{D}} = \frac{{\textbf{c}}^i_{\rm{D}}}{({\textbf{H}}_{\rm{D}}^k\mathbf{B}_{\rm{D}})^T {\textbf{1}}} ({\textbf{H}}_{\rm{D}}^k\mathbf{B}_{\rm{D}})^T \frac{{\textbf{m}}}{{\textbf{H}}_{\rm{D}}^k {\textbf{B}}_{\rm{D}}{\textbf{c}}^i_{\rm{D}}}. \end{aligned}$$

The **h-update** can be solved using the conventional MLEM algorithm w.r.t. the generating function $\mathbf{h}_{\rm{D}}$ (index *j*)

10 $$\begin{aligned} {\textbf{h}}^{j+1}_{\rm{D}} = \frac{{\textbf{h}}^j_{\rm{D}}}{{({\textbf{B}}_{\rm{D}} {\textbf{c}}_{\rm{D}}^{k+1})^T}{\textbf{1}}}(\mathbf{B}_{\rm{D}}{\textbf{c}}_{\rm{D}}^{k+1})^T\frac{{\textbf{m}}}{({\textbf{B}}_{\rm{D}}{\textbf{c}}^{k+1}_{\rm{D}}){\textbf{h}}^j_{\rm{D}}}, \end{aligned}$$

where the non-negativity constraint on the generating functions ${\textbf{h}}_{\rm{D}}$ was implicitly bundled with the MLEM update. The tracer-specific spectral coefficients and generating functions were then extracted from the final estimated ${\hat{{\textbf{c}}}}_{\rm{D}}$ and ${\hat{{\textbf{h}}}}_{\rm{D}}$, respectively, and further used to recover the single-tracer TACs of each voxel in an image via the single-tracer linear model (1). We note that the proposed alternating update scheme for solving (5) without regularisation, i.e., ${\mathcal {R}}=0$, is equivalent to an image-space dual-tracer version of the fully 4D PET image reconstruction algorithm (IS-F4D), described in Appendix A. In this case, the **c-update** only contains the MLEM step w.r.t. the spectral coefficients ${\textbf{c}}_{\rm{D}}$ [42].

We note further that model (5) leads to a non-smooth, bi-convex optimization problem in ${\textbf{c}}_{\rm{D}}$ and ${\textbf{h}}_{\rm{D}}$ (assuming ${\mathcal {R}}$ are closed proper convex), i.e., it is convex in each of the variables ${\textbf{c}}_{\rm{D}}$ and ${\textbf{h}}_{\rm{D}}$ when the other is considered fixed, but non-convex as a function of both variables. Therefore, the global convergence of the proposed alternating update scheme is not guaranteed, and the solution of the minimization problem is non-unique (even without regularisation [20]), due to their use in ${\hat{{\textbf{h}}}_{\rm{D}}\textbf{B}_{\rm{D}}{\hat{\textbf{c}}}_{\rm{D}}}$ minimizing the objective function in (5).

### Kinetic model-informed deep network for dual-tracer PET image separation

The vast majority of the DL-based methods for mPET image separation are purely data-driven with a very weak inductive prior. With the recent success of unrolled deep networks for PET reconstruction [30, 31, 45–49], we improved the inductive prior of the deep network by unrolling the alternating update scheme presented in Section "Fully 4D inspired dual‑tracer PET image separation", and replaced the proximal step in each iteration with a trained block-dependent convolutional neural network (CNN) to form the proposed kinetic model-informed deep network for dual-tracer PET image separation. To sufficiently accelerate the separation process, we use one update with Eqs. (9) and (8), and one update with Eq. (10), leading to the following update scheme

11 $$\begin{aligned} \begin{aligned} \bar{{\textbf{c}}}^k_{\rm{D}}&= \frac{{\textbf{c}}^k_{\rm{D}}}{({\textbf{H}}_{\rm{D}}^k\mathbf{B}_{\rm{D}})^T {\textbf{1}}} ({\textbf{H}}_{\rm{D}}^k\mathbf{B}_{\rm{D}})^T \frac{{\textbf{m}}}{{\textbf{H}}_{\rm{D}}^k {\textbf{B}}_{\rm{D}}{\textbf{c}}^k_{\rm{D}}} \\ {\textbf{c}}_{\rm{D}}^{k+1}&= \Gamma _{\theta _k} (\bar{{\textbf{c}}}^k_{\rm{D}})\\ {\textbf{h}}^{k+1}_{\rm{D}}&= \frac{{\textbf{h}}^k_{\rm{D}}}{{({\textbf{B}}_{\rm{D}} {\textbf{c}}_{\rm{D}}^{k+1})^T}{\textbf{1}}}(\mathbf{B}_{\rm{D}}{\textbf{c}}_{\rm{D}}^{k+1})^T\frac{{\textbf{m}}}{({\textbf{B}}_{\rm{D}}{\textbf{c}}^{k+1}_{\rm{D}}){\textbf{h}}^k_{\rm{D}}}, \end{aligned} \end{aligned}$$

where $\Gamma _{\theta _k}: {\mathbb {R}}^{J(E+F)\times 1}_{\ge 0}\rightarrow {\mathbb {R}}^{J(E+F)\times 1}_{\ge 0}$ corresponds to a trained CNN in the *k*th iteration (or the *k*th block in the kinetic model-informed unrolled deep network) with block-dependent trainable parameters $\theta _k$.

**Fig. 2:**
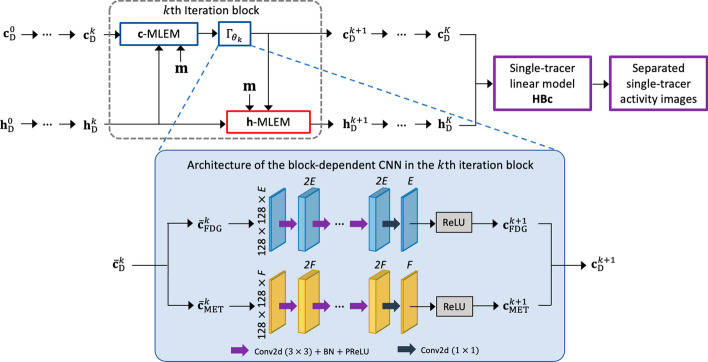
The proposed kinetic-model informed deep network architecture, wher *E* and *F* are the number of the predefined exponential temporal basis function of [$^{18}$F]FDG and [$^{11}$C]MET, respectively, and *K* is the number of iteration blocks. In each iteration block, the blue and red boxes perform a one-step MLEM update w.r.t. the spectral coefficients ${\textbf{c}}_{\rm{D}}$ and the generating functions ${\textbf{h}}_{\rm{D}}$, respectively. The information shown in the figure is based on the simulation study for dual-tracer [$^{18}$F]FDG+[$^{11}$C]MET PET image separation described in Section "Simulation and validation"

The architecture of the proposed kinetic model-informed unrolled deep network based on the update scheme (11) with *K* iteration blocks is demonstrated in Fig. 2. In the *k*th iteration block, the spectral coefficients ${\textbf{c}}^k_{\rm{D}}$ were first updated via an MLEM step. The updated coefficients were then fed into a trained block-dependent CNN, i.e., $\Gamma _{\theta _k}$, to perform the proximal update (or regularisation). The network $\Gamma _{\theta _k}$ consists of two branches, one for each tracer. Each branch consists of the repeated application of several multi-channel $3\times 3$ 2D convolutional layers, each followed by a batch normalization (BN) and a parametric rectified linear unit (PReLU), as well as a $1\times 1$ 2D convolutional layer at the end. In addition, we activated the output layer using a ReLU to enforce the non-negativity constraint on the spectral coefficients. The generating functions ${\textbf{h}}^k_{\rm{D}}$ were then updated via a one-step MLEM with use of the output from $\Gamma _{\theta _k}$, i.e., ${\textbf{c}}^{k+1}_{\rm{D}}$. After the *K*th iteration block, the single-tracer linear model (1) recovers the separated single-tracer activity images from the final estimated decay coefficients ${\textbf{c}}^{K}_{\rm{D}}$ and generating functions ${\textbf{h}}^{K}_{\rm{D}}$.

## Simulation and validation

### Simulation setup

**Fig. 3:**
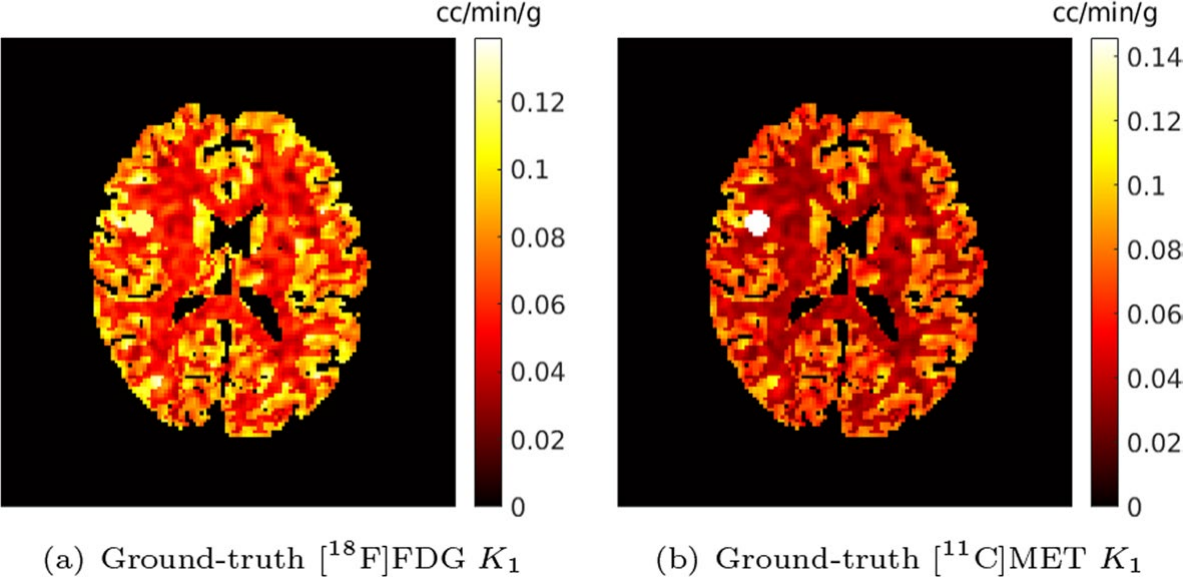
An example of a simulated ground-truth parametric map $K_1$ for (a) [$^{18}$F]FDG and (b) [$^{11}$C]MET

We simulated the tracer activity images of [$^{18}$F]FDG and [$^{11}$C]MET using a 2D BrainWeb phantom dataset. 5 non-contiguous slices were selected from each of the 20 3D BrainWeb phantoms^1^ [50]. 100 slices were obtained in total to form the 2D BrainWeb phantom dataset. Each of the 2D brain phantom contains white matter (WM) and grey matter (GM), and is of resolution $128\times 128$ with a voxel size of $2.602\times 2.602$ mm$^2$. A tumour (TM) with diameter ranging between 12 and 18 mm and randomly located was added to each 2D phantom. To generate the ground-truth parametric maps of each tracer, the kinetic parameters for a given region were sampled from a Gaussian distribution with mean values based on those from the literature [17], as listed in Table 1, and with the coefficient of variation equal to 0.1 (absolute values were taken after sampling). Randomised structures were then introduced to the white matter and grey matter regions to simulate heterogeneous variation within the whole brain using the BrainWeb library [51]. An example of the simulated ground-truth parametric maps $K_1$ for FDG and MET are shown in Fig. 3. The AIFs of each tracer were generated along with the shape of the AIFs from the literature [17] using Feng’s input function model [52]. Feng’s input function parameters were also modeled as Gaussian variables with the coefficient of variation equal to 0.1 (the absolute values were taken after sampling), to simulate the population variation in the dataset [37, 40].

**Table 1:** Mean values of the kinetic parameters of tissue ROIs

	FDG				MET				
	$\user2{K}_{{\mathbf{1}}}$	$\user2{K}_{{\mathbf{2}}}$	$\user2{K}_{{\mathbf{3}}}$	$\user2{V}_{{\mathbf{B}}}$	$\user2{K}_{{\mathbf{1}}}$	$\user2{K}_{{\mathbf{2}}}$	$\user2{K}_{{\mathbf{3}}}$	$\user2{K}_{{\mathbf{4}}}$	$\user2{V}_{{\mathbf{B}}}$
WM	0.05	0.11	0.05	0.026	0.04	0.06	0.04	0.028	0.026
GM	0.10	0.14	0.17	0.103	0.08	0.08	0.10	0.017	0.103
TM	0.11	0.10	0.15	0.173	0.13	0.03	0.06	0.012	0.173

Units: $K_1$: cc/min/g; $k_2-k_4$: min$^{-1}$; $V_B$: unitless

**Fig. 4:**
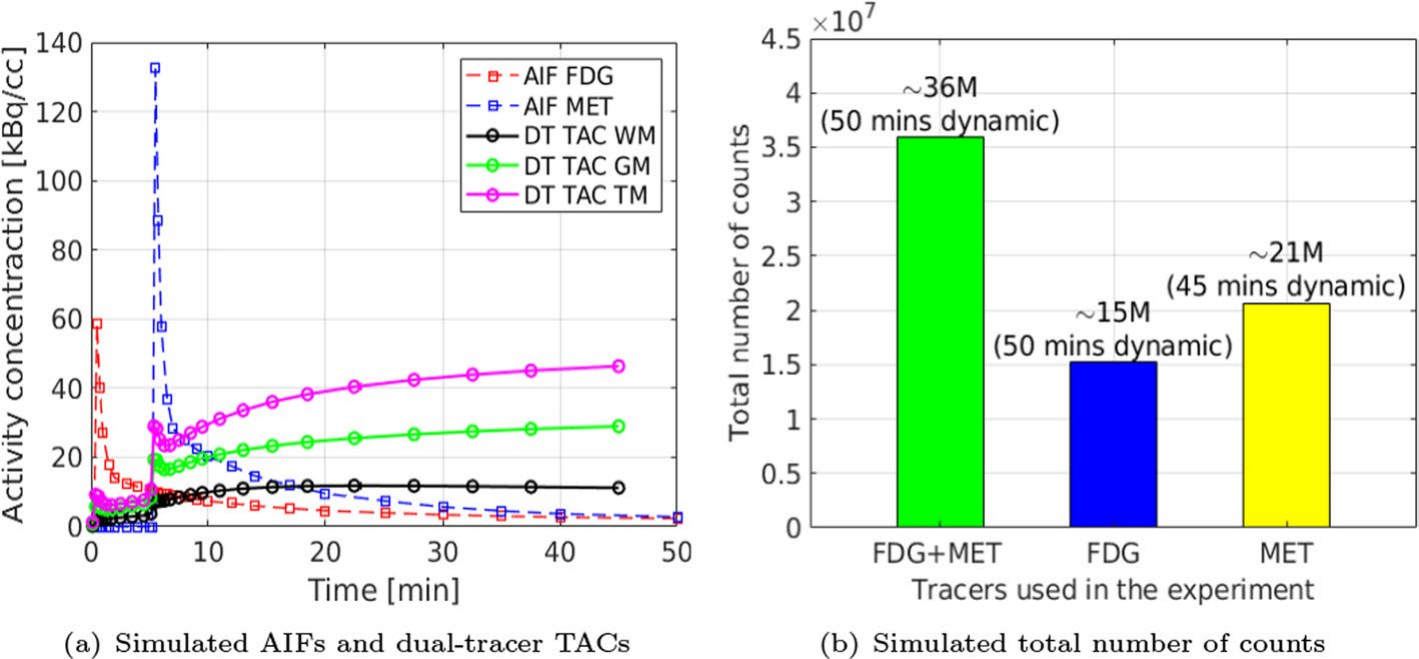
A simulated example of (a) AIFs (dashed lines) and dual-tracer TACs for each tissue ROIs, and (b) total number of counts in a dynamic dual-tracer scan and dynamic single-tracer scans

The single-tracer voxel-wise TACs (activity images) were generated from the simulated ground-truth parametric maps using the irreversible two-tissue compartment model for FDG, and reversible two-tissue compartment model for MET, respectively, following a similar approach to that described in [37]. Note that fractional blood volume $V_B$ was also included in the simulation. Radioactive decay of each tracer was also modeled in the simulation, which requires the integration of decay constants $\lambda _{[{}^{18}\text {F}]} = \log (2)/109.8$ min$^{-1}$ and $\lambda _{[{}^{11}\text {C}]} = \log (2)/20.4$ min$^{-1}$. The single-tracer activity images then were summed up together to form the ground-truth dual-tracer activity images. We followed a dynamic dual-tracer PET scanning protocol proposed in [17, 37] for [$^{18}$F]FDG+[$^{11}$C]MET, which was conducted for 50 mins, with [$^{11}$C]MET injected 5 mins after [$^{18}$F]FDG injection. The dynamic PET scan (dual-tracer or single-tracer) was divided into 27 time frames: $4\times 0.25$ mins, $2\times 0.5$ mins, $3\times 1$ mins, $4\times 0.25$ mins, $2\times 0.5$ mins, $3\times 1$ mins, $2\times 2$ mins, $2\times 3$ mins, $4\times 5$ mins, $1\times 10$ mins. An example of the simulated AIFs and dual-tracer (DT) TACs without decay correction within each ROIs is shown in Fig. 4(a).

For reconstructions of the simulated data, we modeled a GE Discovery ST PET-CT scanner with system sensitivity ${\sim }$2 cps/kBq in 2D mode [53]. Ground-truth dual-tracer and single-tracer activity images were first forward projected to generate noise-free sinogram data based on the aforementioned dynamic scan protocol using a pre-calculated system matrix. An attenuation map was simulated with a constant linear attenuation coefficient assgined in the whole brain. We follow a similar setup as described in [37, 40], where a $20\%$ uniform background was included to account for scatter and random events. A scaling factor was applied to this projected data in order to generate the mean-count sinogram, prior to the introduction of Poisson noise into each sinogram bin. This scaling factor was chosen so as to obtain datasets containing a predetermined expectation of the total counts in each sinogram, where the expectation of total counts was modelled based on the system sensitivity of the scanner (see Fig. 4(b) for example). Dynamic images for both dual-tracer and single-tracer acquisitions were then reconstructed frame by frame using the MLEM algorithm (initialised by uniform images) with 128 iterations without post-smoothing. Reconstructed images were frame-length corrected reconstructed tracer activity images.^2^

### Implementation details and reference methods

Four separation methods were compared in this study: (1) the conventional voxel-wise MTCM (v-MTCM [11]) method, (2) the image-space dual-tracer version of the fully 4D method (IS-F4D; see earlier Section "Fully 4D inspired dual‑tracer PET image separation" and Appendix A), (3) the pure-data-driven convolutional encoder-decoder (CED) [37], and (4) the proposed kinetic-informed unrolled deep network.

The v-MTCM method estimates the single-tracer activity images by fitting the dual-tracer kinetic model to the measured dual-tracer activity images (dynamic dual-tracer noisy MLEM reconstruction) using the voxel-wise weighted least squares (VWLS) with a known AIF of each tracer. In our study, we focused on the validation of the algorithm and thus assumed the AIFs of each tracer to be known in v-MTCM. The weighting factors used were the time frame durations in order to compensate for non-uniform temporal sampling [12]. Note that decay correction can be accounted for after separation because the proportion of each tracer (and hence its decay correction factor) is unknown beforehand [13]. Therefore, radioactive decay is not taken into account in the weighting factors. The v-MTCM method first estimates the parametric images of each tracer in the separation process. The estimated single-tracer parametric images (with the tracer-specific AIFs) were then used for recovering the single-tracer activity images. The trust-region-reflective algorithm was used to perform the VWLS fitting. Stopping criteria were set such that the optimization procedure terminates when the relative error of the estimate was less than $1\times 10^{-8}$ or the maximum iteration number (1600 iterations) was achieved. The initial values of the kinetic parameters were set to be 0.01 for all voxels. The lower bounds of each parameter were set to be $1\times 10^{-11}$ and the upper bounds of $V_B$, $K_1$ and $k_2-k_4$ were set to be [1, 5, 2, 1, 1, 1], respectively.

The IS-F4D method separates the dual-tracer activity images (dynamic dual-tracer noisy MLEM reconstruction) without the use of AIFs. This is achieved by estimating the spectral coefficients and the generating functions in an alternating manner. A set of predetermined exponential temporal basis functions for [$^{18}$F]FDG and [$^{11}$C]MET are shown in Fig. 1. The number of the exponential temporal basis function were chosen to be $E = F = 25$, and the decay constants were logarithnmically spaced between 0.001 and 5 min$^{-1}$ as suggested in [20, 42], including the special case of the decay constant equal to zero (more details on the impact of the number of exponentials for representing TACs in spectral analysis can be found in [42]). Both the spectral coefficients and the generating functions were initialised to vectors containing ones [20]. An initial MLEM 16 updates (indexed by *i*) was used to solve the **c-update** (with ${\mathcal {R}} = 0$), followed by a repeated cycle of: 4 MLEM updates for the **c-update** (${\mathcal {R}} = 0$, indexed by *i*) and 1 MLEM update for the **h-update** (indexed by *j*) as suggested in [42]. A total 1600 iterations were used in the outer iteration (indexed by *k*). Note that, in each MLEM update, we resampled the measured dual-tracer activity images (dual-tracer voxel-wise TACs) and the estimated generating functions of each tracer into finer samples to accommodate the convolution with each of the time functions resulting from $\textbf{Bc}$.

Both the pure data-driven CED and the proposed kinetic model-informed unrolled deep network can separate the dual-tracer dynamic images without the use of AIFs. For network training, dynamic dual-tracer [$^{18}$F]FDG+[$^{11}$C]MET noisy MLEM reconstruction was used as the network input and dynamic single-tracer noisy MLEM reconstructions were used as training labels. The mean square error (MSE) loss is applied to the activity images of each tracer, and their sum is used as the loss function for network training, which is given as

12 $$\begin{aligned} {\mathcal {L}} = \sum _{n\in \{\text {FDG},\text {MET}\}} \biggl \{\frac{1}{N_S}\sum _{s=1}^{N_S}||\mathring{C}^{(n)}_{s} - {\hat{C}}^{(n)}_{s}||_2^2 \biggl \}, \end{aligned}$$

where $\mathring{C}^{(n)}$ denotes the separated single-tracer activity images of the *n*th tracer in the network output, ${\hat{C}}^{(n)}$ is the label single-tracer activity images, and $N_S$ is the total number of training pairs. The proposed deep network contains $K=10$ iteration blocks (due to GPU memory limitations) and each block-dependent CNN contains 3 convolution layers ($3\times 3$ 2D convolution). The same exponential temporal basis functions as IS-F4D were used in the proposed deep network. Both the spectral coefficients and the generating functions were again initialised to vectors containing ones. For a fair comparison, we adjusted the number of channels in the CED presented in [37] to match the number of trainable parameters in the proposed deep network. The number of trainable parameters for both networks is around 1.2 million. All network training was performed in the same manner. We note that the proposed deep network was trained end-to-end. For network training, we used 80 simulated data examples generated from 16 BrainWeb phantoms. An additional 10 examples, generated from two other BrainWeb phantoms, were allocated for validation. The 10 examples used for testing were generated from the remaining 2 BrainWeb phantoms. The network parameters were initialised using the Xavier initialisation. The Adam algorithm was used with a learning rate $1\times 10^{-4}$ and a batch size equal to 8 for network training. All networks were trained for a maximum of 1200 epochs with early stopping when there were no improvements in the validation metrics. The training and evaluation steps were implemented in PyTorch, on an PC with a NVIDIA GeForce RTX 3090 GPU.

### Evaluation metrics

The quality of the separated single-tracer activity images was evaluated over $R=20$ different noise realisations using the voxel-level normalized root mean square error (NRMSE)

13 $$\begin{aligned} \rm{NRMSE} = \sqrt{\rm{Bias}^2 + \rm{SD}^2}, \end{aligned}$$

with the bias and the standard deviation (SD) given by [54]

14 $${\text{Bias}} = \sqrt {\frac{{\sum\limits_{{l \in \Omega }} {(\bar{x} - x_{l}^{{{\text{Ref}}}} )^{2} } }}{{\sum\limits_{{l \in \Omega }} {(x_{l}^{{{\text{Ref}}}} )^{2} } }}} \times 100\% ,\,\,\,{\text{SD }} = {\text{ }}\sqrt {\frac{1}{R}\frac{{\sum\limits_{{r{\text{ }} = {\text{ }}1}}^{R} {\sum\limits_{{l \in \Omega }} {(\bar{x}_{l} - x_{l}^{r} )^{2} } } }}{{\sum\limits_{{l \in \Omega }} {(x_{l}^{{{\text{Ref}}}} )^{2} } }}} \times 100\% ,$$

where $\Omega$ is the whole brain region, $\bar{x}_l = \frac{1}{R}\sum _{r=1}^R x^r_l$ is the mean value for voxel *l* in the separated image ${\varvec{x}}$, found by taking the average of the *R* noise realisations, and ${\varvec{x}}^{\rm{Ref}}$ is a reference image for error calculation. In our study, single-tracer noise-free (NF) MLEM reconstructions (initialised by uniform images, with 128 iterations) was used as the reference image in all cases.

TACs extracted from the tumour ROIs in the separated single-tracer activity images were also used to validate the separation performance at the ROI level. The NRMSE of the TACs was calculated to evaluate the tumour ROI-TAC quantification

15 $$\begin{aligned} \rm{NRMSE}_{\rm{TAC}}^{\rm{TM}} = \sqrt{{\rm{Bias}_{\rm{TAC}}^{\rm{TM}}}^2 + {\rm{SD}_{\rm{TAC}}^{\rm{TM}}}^2}, \end{aligned}$$

with the bias and SD given by

16 $${\text{Bias}}_{{\text{TAC}}}^{\text{TM}} \, =\,\frac{{|\bar{c} - c^{{{\text{Ref}}}} |}}{{c^{{{\text{Ref}}}} }} \times 100\% ,\,\,\,\,{\text{SD}}_{\text{TAC}}^{\text{TM}} {\text{ }} = {\text{ }}\frac{1}{{c^{{{\text{Ref}}}} }}\sqrt {\frac{1}{R}\sum\limits_{{r{\text{ }} = {\text{ }}1}}^{R} {(c^{r} - \bar{c})^{2} } } \times 100\% ,$$

where $c^{\rm{Ref}}$ is the single-tracer ROI TACs extracted from the tumour regions in the dynamic single-tracer noise-free MLEM and $\bar{c} = \frac{1}{R}\sum _{r=1}^R c^r$ denotes the mean of *R* noise realisations, and $c^r$ is the tumour ROI TACs with the mean ROI uptake in each time frame in the *r*th realisation.

## Results

### Separated image quality

**Fig. 5:**
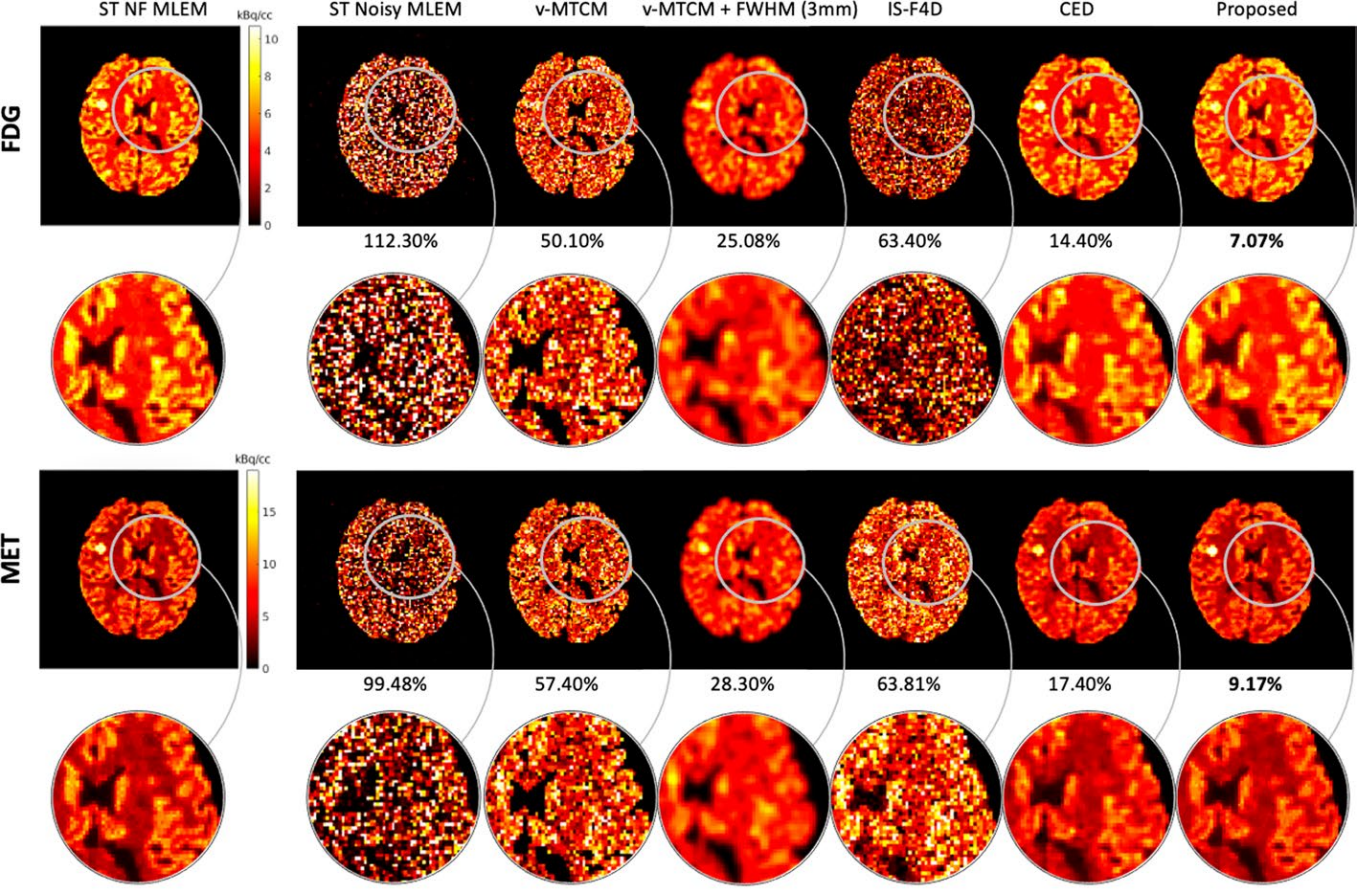
Frame 14 ($t = 6.25$ min) of the separated tracer activity images for FDG and MET in a test example using v-MTCM, v-MTCM+FWHM (3 mm), IS-F4D, the CED [37], and the proposed kinetic model-informed unrolled deep network. NRMSE values of each separated image are shown at the bottom. For plotting only, we limit the colour scale of the figures to fall within the same range as the reference images shown in the first column

Figure 5 shows the reference activity images (see column 1) and the separated images by using different methods for frame 14 (an early 30-s frame at 1.25 min after the [$^{11}$C]MET injection) in a test example. The single-tracer noisy MLEM suffers from visually-evident high noise. The model-based methods, v-MTCM and IS-F4D, enable the suppression of noise compared to the results obtained from the single-tracer noisy MLEM. By introducing a 3 mm full-width at half maximum (FWHM) Gaussian kernel on the separated images obtained from v-MTCM, denoted as v-MTCM+FWHM (3 mm), we obtained images with reduced noise but excessive smoothing. The DL-based methods (the CED and the proposed kinetic model-informed unrolled deep network) achieved better image quality than the model-based methods. By incorporating the general kinetic model into the unrolled deep network, the proposed method enhances the recovery of the detailed structures in the grey matter and white matter regions compared to the pure data-driven CED (see the zoomed-in patches shown in Fig. 5 for comparison).

**Fig. 6:**
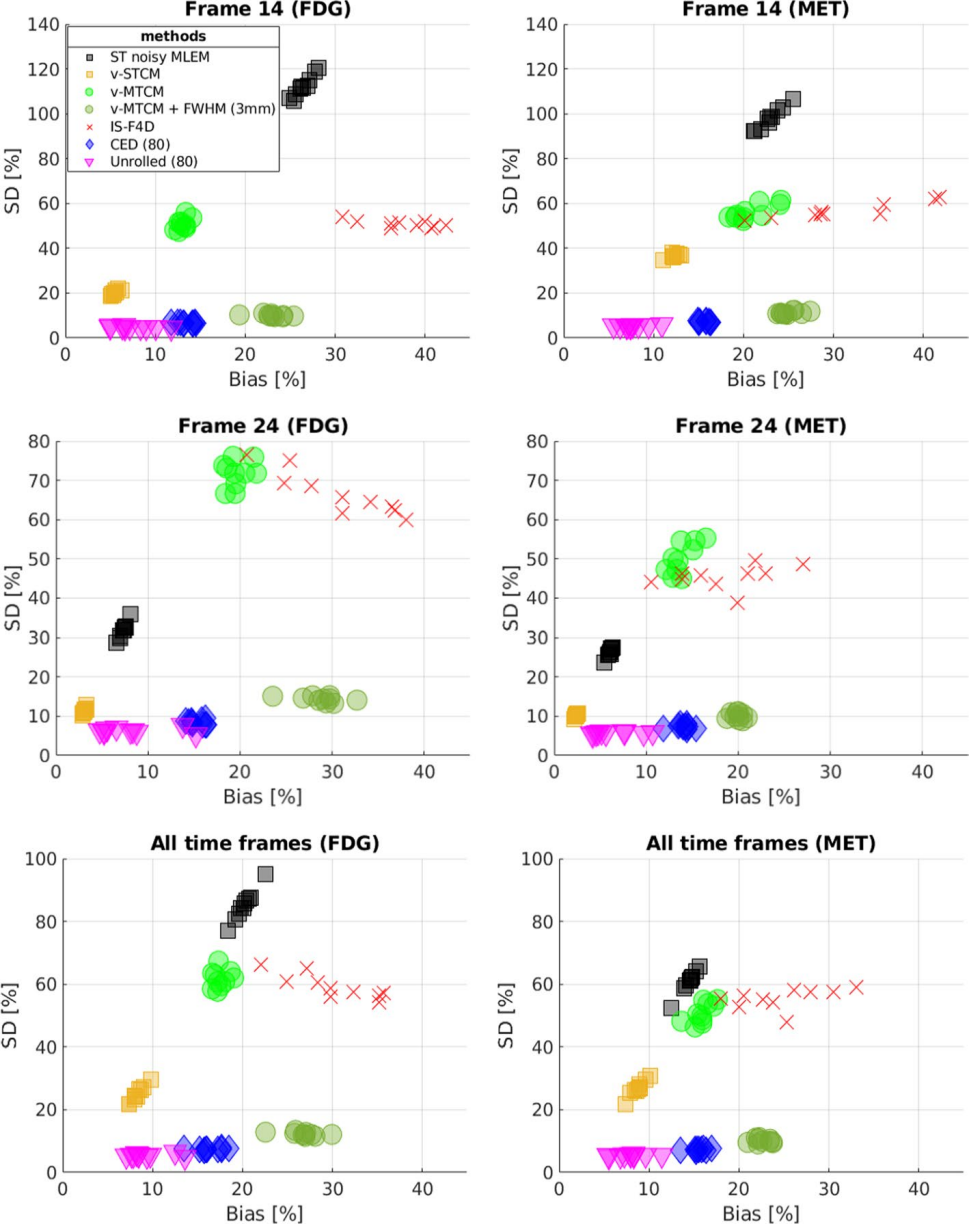
Plots of bias-SD trade-off (over 10 test examples, each with 20 different noise realisations) for the separated single-tracer activity images using different methods for frame 14 (row 1), frame 24 (row 2), and the whole activity image sequences (row 3) for FDG and MET, respectively

Figure 6 shows the trade-off between the bias and SD of different methods for frame 14, frame 24 (a later 5-min frame at 27.5 min after [$^{11}$C]MET injection), and the separated single-tracer activity image sequences (all time frames were considered), over the entire test dataset (10 test examples). For the early time frame (frame 14), on average, the single-tracer noisy MLEM achieved ${\sim }25\%$ bias and ${\sim }110\%$ SD for FDG, and ${\sim }25\%$ bias and ${\sim }100\%$ SD for MET, respectively (see Fig. 6(row 1)). While fitting the single-tracer compartment model (with the simulated tracer-specific AIFs) to the single-tracer noisy MLEM voxel by voxel, denoted as v-STCM (with frame durations as weighting factors), the bias and SD were reduced to ${\sim }5\%$ and ${\sim }20\%$ for FDG, and ${\sim }11\%$ bias and ${\sim }30\%$ for MET, which were highlighted in yellow (also see the black clusters for a comparison). This observation shows that fitting the single-tracer compartment model to the single-tracer noisy MLEM helps to improve the image quality in the early time frame. However, the situation worsens when dual-tracer separation is considered. By fitting the combined compartment model to the dual-tracer noisy MLEM, v-MTCM failed to achieve similar bias and SD levels as those of v-STCM. The main reason is that v-MTCM requires a long delayed time interval between two tracer injections, and it is susceptible to noise and prone to fall into local minima. Post-smoothing with a 3 mm FWHM Gaussian kernel can effectively reduce the SD levels, albeit at the cost of an increase in bias compared to the results obtained from v-MTCM. Another model-based method, IS-F4D, achieved a similar SD level but exhibited higher bias compared to v-MTCM due to the non-unique solution of the optimization problem. For the DL-based methods, the pure data-driven CED reduced the SD values by ${\sim }40\%$ in the early time frame compared to the model-based methods, and the SD values are also lower than those of v-STCM. The CED uses spatiotemporal information for separation while the model-based methods only use temporal information for separation. In addition, the CED with MSE loss learns to output the mean of all plausible noisy explanations when it is trained using noisy labels, and thus the CED implicitly learns to denoise the separated images [37]. However, no significant improvement in bias was found using the CED for FDG, and only a small improvement was observed for MET, compared to v-MTCM. The proposed kinetic model-informed unrolled deep network outperformed the aforementioned methods with additional debiasing compared to the CED. It delivered approximately a $2\%$ higher bias for FDG and a $4\%$ lower bias for MET in the early time frame, compared to the results obtained from single-tracer noisy data using v-STCM.

Similar results were found in the later time frame, where v-MTCM+FWHM (3 mm) and the DL-based methods achieved much lower SD compared to v-MTCM and IS-F4D (see Fig. 6(row 2)). The bias level of the CED is similar for MET and improved for FDG, compared to those of the v-MTCM method. Although the proposed method further reduced the bias compared to the CED, it is still higher than that of v-STCM. This indicates that the separation task remains challenging for our proposed method in the later time frames (relatively high-count compared to the early time frame), especially given that only 80 data examples were used for network training.

The overall separation performance of the single-tracer activity image sequences is shown in Fig. 6(row 3), where it again shows that the DL-based method achieved much lower SD compared to the model-based methods. The proposed deep network outperformed the CED by further reducing the bias and SD values. On average, the proposed method achieved a similar bias level and lower SD for both FDG and MET, compared to those that would be obtained with single-tracer data using v-STCM.

### Impact of the number of training examples

**Fig. 7:**
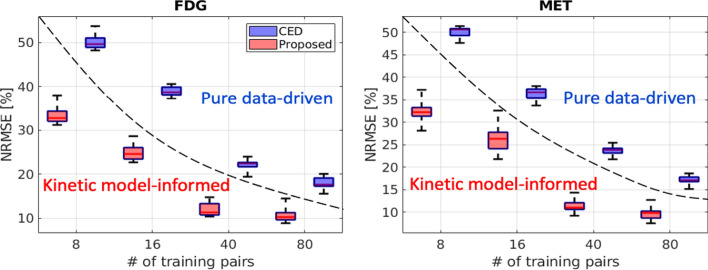
NRMSE of the separated single-tracer activity image sequences (over 10 test examples, each with 20 different noise realisations) using the pure data-driven CED and the proposed kinetic model-informed unrolled deep network with different number of training examples

We retrained the pure data-driven CED and the proposed kinetic-model informed unrolled deep network using different sample sizes to assess the impact of the number of training examples for dual-tracer [$^{18}$F]FDG+[$^{11}$C]MET PET image separation. Figure 7 shows that the NRMSE values of the separated single-tracer activity image sequences decrease as the number of training examples increases (from 8 to 80) for both the CED and the proposed kinetic model-informed deep network. On average, the CED dual-tracer separation using 80 training examples achieved ${\sim }18\%$ and ${\sim }17\%$ NRMSE for FDG and MET, respectively. Compared to the CED, the proposed deep network reduced the NRMSE values by ${\sim }8\%$ and ${\sim }7\%$ using 80 training examples. When fewer training examples are considered, the proposed deep network still achieves lower NRMSE compared to the pure data-driven CED. This indicates that incorporating the kinetic model into the unrolled deep network forms a stronger inductive prior, leading to a better separation performance with less training data.

### Parametric map estimation

Parametric imaging was also performed for the separated single-tracer activity images. We note that v-MTCM, by definition, directly estimates the parametric images of each tracer in the separation process, eliminating the need for post-estimation. To have a fair comparison, the v-STCM method was used to estimate the parametric maps from the separated single-tracer activity images. For v-MTCM+FWHM (3 mm), post-smoothing was applied to the separated images after v-MTCM. Therefore, the parametric images for each tracer were re-estimated by applying v-STCM to the separated post-smooth images. The parametric maps recovered from the single-tracer noise-free MLEM were used as reference images.

**Fig. 8:**
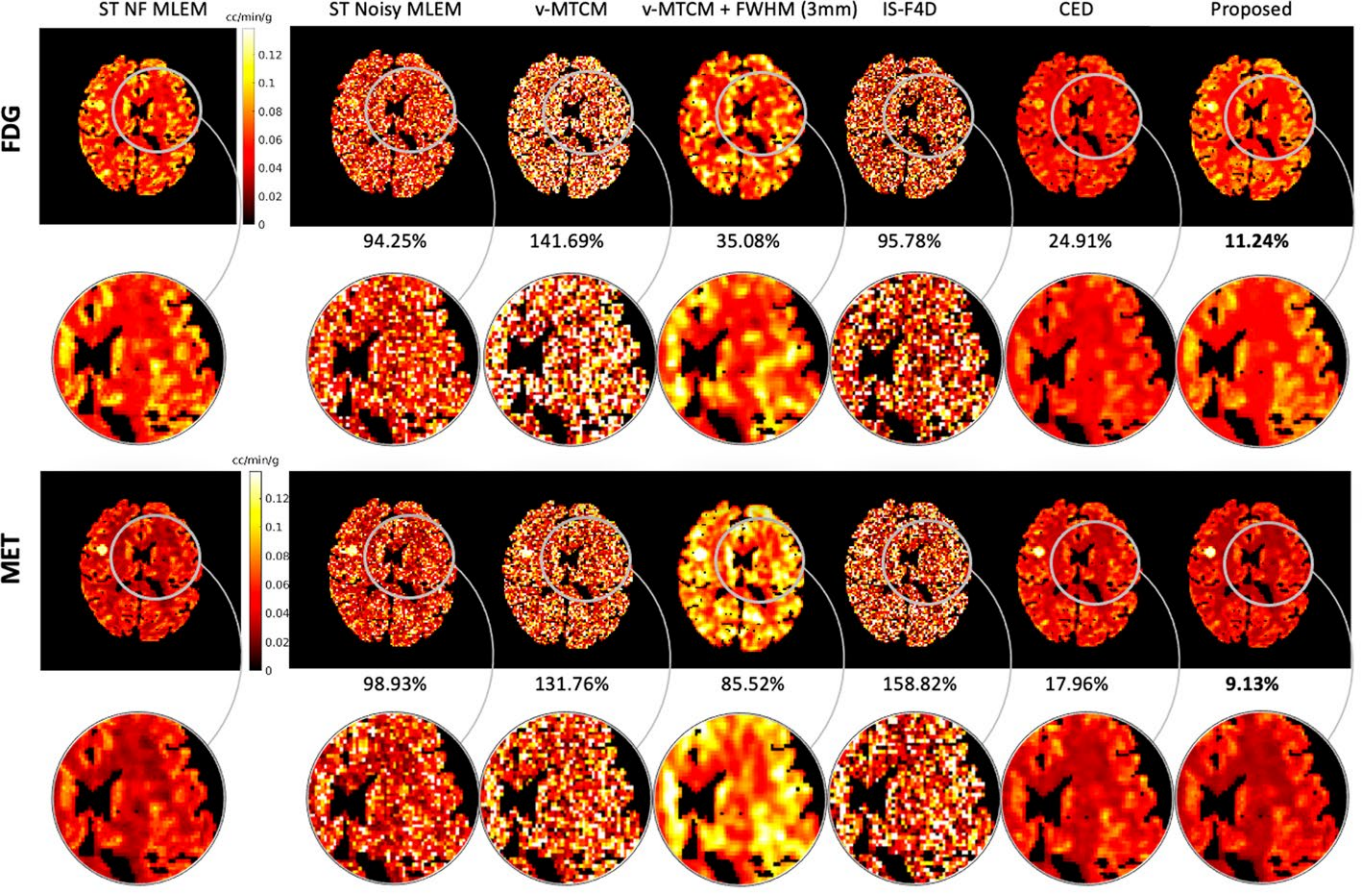
A test example of the parametric maps $K_1$ estimated from the separated single-tracer activity images for FDG and MET, respectively (also see Fig. 3 for the ground-truth $K_1$ images). NRMSE values of each estimated $K_1$ image were shown at the bottom. For plotting only, we limit the colour scale of the figures to fall within the same range as the reference images shown in the first column

Figure 8 shows a test example of the separated parametric images $K_1$ for [$^{18}$F]FDG and [$^{11}$C]MET (also see Fig. 3 for the ground-truth $K_1$ images). Compared to the single-tracer noisy MLEM, the separated parametric image $K_1$ for both FDG and MET obtained by v-MTCM and IS-F4D are much noisier. The $K_1$ images estimated using v-MTCM with a 3 mm FWHM post-smoothing were much less noisy but excessively smoothed. The CED substantially reduced the noise but also led to the loss of some detailed structures within the gray and white matters. In comparison, the $K_1$ images obtained by the proposed kinetic model-informed unrolled deep network retrieved more details in the whole brain regions for both FDG and MET (see the zoomed-in patches shown in Fig. 8 for comparison).

**Fig. 9:**
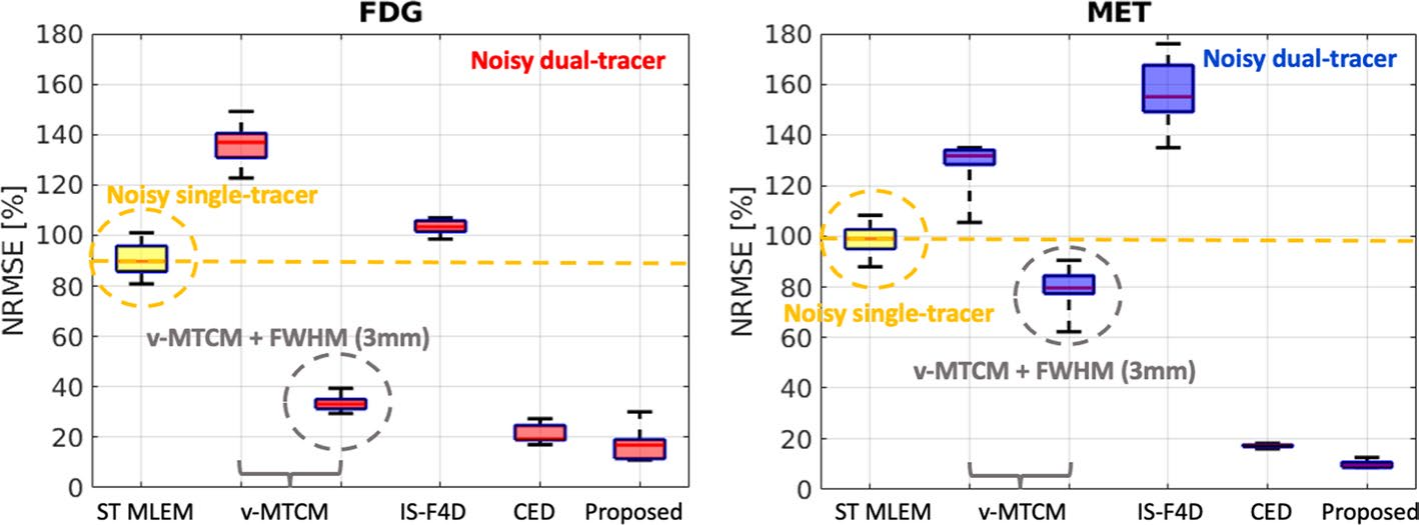
NRMSE of the parametric maps $K_1$ (over 10 test examples, each with 20 different noise realisations) estimated from the separated single-tracer activity images for FDG and MET, respectively. The yellow dashed lines indicate the median of the boxplots for the parametric maps $K_1$ estimated from the single-tracer noisy MLEM activity images

Figure 9 further shows a quantitative comparison of different methods for the separated FDG and MET $K_1$ images. On average, ${\sim }90\%$ and ${\sim }100\%$ NRMSE were found in the estimated $K_1$ images using v-STCM. Compared to v-STCM, v-MTCM and IS-F4D achieved higher NRMSE for both FDG and MET, which is consistent with the visual observation in Fig. 8. The v-MTCM+FWHM (3 mm) method effectively reduced the NRMSE values, mainly due to the denoising effect of post-smoothing. Notably, the CED outperformed the two model-based methods and ST MLEM with ${\sim }20\%$ NRMSE for both FDG and MET. Again, the CED with MSE loss learns to output the mean when trained using noisy labels, implicitly denoising the separated images. As a consequence, we obtained better estimates of $K_1$. The proposed method further reduced the NRMSE values by ${\sim {4}}\%$ (FDG) and ${\sim {10}}\%$ (MET). This is because embedding the kinetic model into the unrolled deep network offers a stronger inductive prior to the deep learning framework compared to the pure data-driven CED, leading to further debiasing and resulting in a superior image separation performance (see Subsection "Separated image quality" for more details). As a consequence, the proposed deep network achieved an even better estimate of $K_1$.

**Fig. 10:**
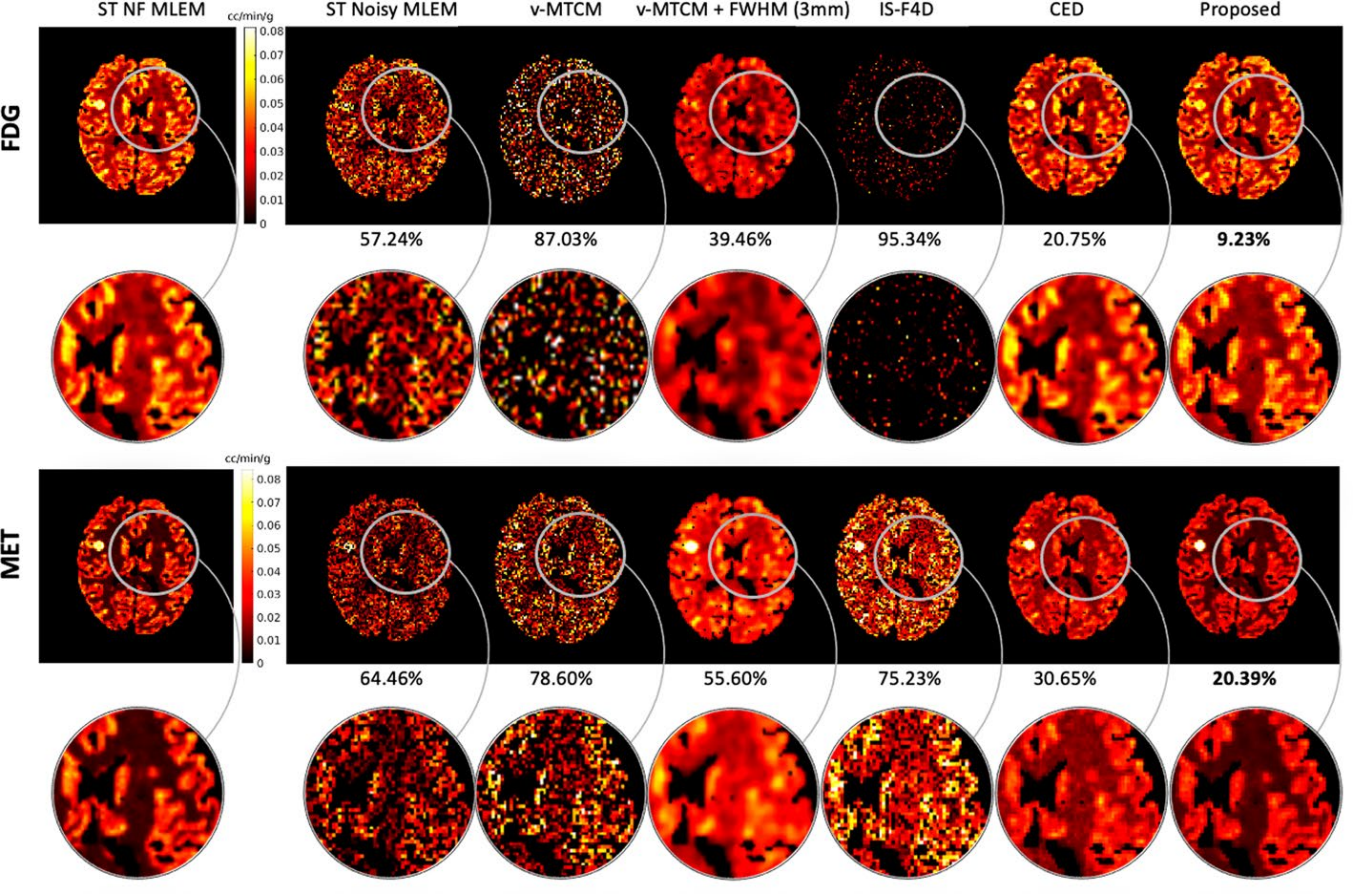
A test example of the parametric maps $K_i$ estimated from the separated single-tracer activity images for FDG and MET, respectively. NRMSE values of each estimated $K_i$ image were shown at the bottom. For plotting only, we limit the colour scale of the figures to fall within the same range as the reference images shown in the first column

**Fig. 11:**
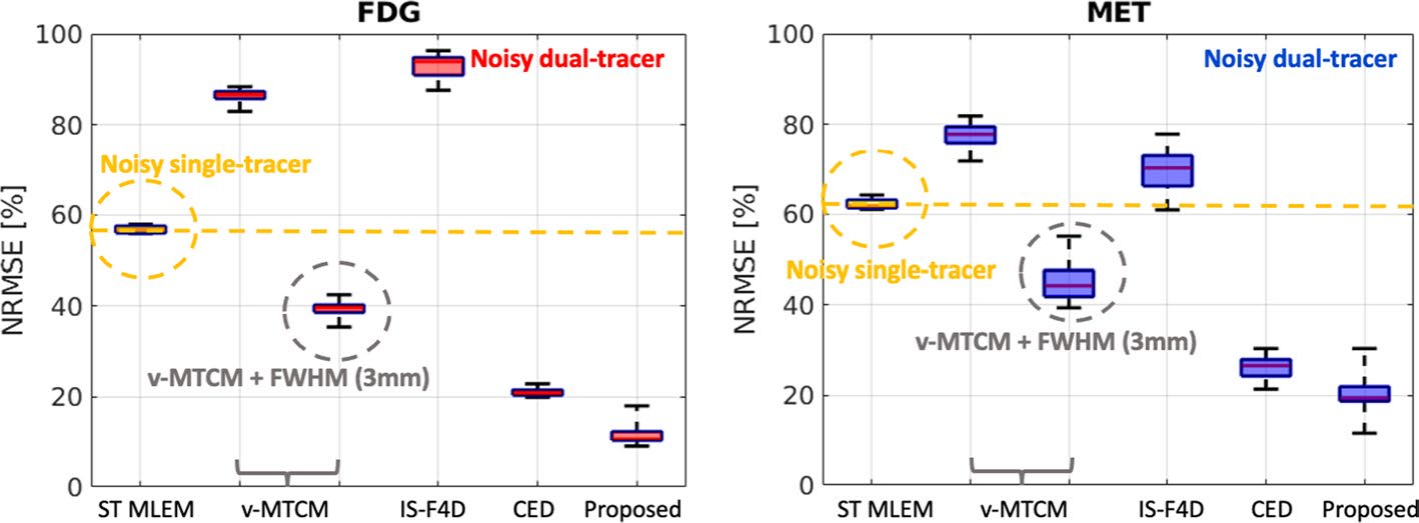
NRMSE of the parametric maps $K_i$ (over 10 test examples, each with 20 different noise realisations) estimated from the separated single-tracer activity images for FDG and MET, respectively. The yellow dashed lines indicate the median of the boxplots for the parametric maps $K_i$ estimated from the single-tracer noisy MLEM activity images

Next, a test example of the separated parametric images $K_i=K_1k_3/(k_2+k_3)$ for [$^{18}$F]FDG and [$^{11}$C]MET is shown in Fig. 10. In comparison, the $K_i$ images obtained by the proposed kinetic model-informed unrolled deep network retrieved more details in the whole brain regions for both FDG and MET compared to other methods (see the zoomed-in patches shown in Fig. 10 for comparison). Figure 11 further shows the NRMSE values of different methods for the separated FDG and MET $K_i$ images over 10 test examples. Similar to the results for $K_1$, the proposed deep network achieved lower NRMSE values compared to the model-based methods and the pure data-driven CED.

### Separation of tumour ROI TACs

**Fig. 12:**
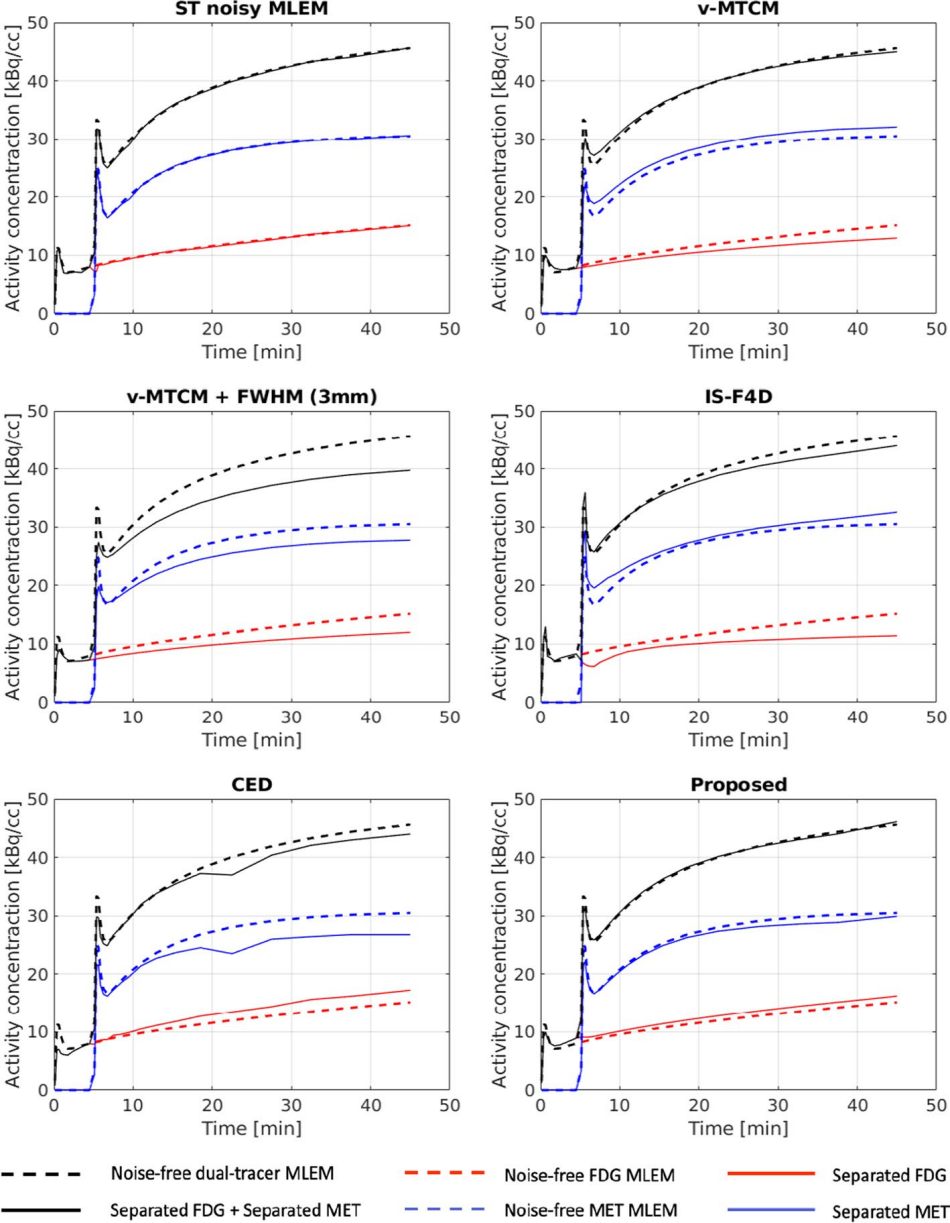
A test example of mean TACs (over 20 noise realisations) extracted from the tumour ROI in the separated single-tracer activity images for FDG and MET, respectively

A test example of the tumour ROI TACs (averaged over 20 different noise realisations) extracted from the separated single-tracer activity images using different separation methods is demonstrated in Fig. 12. Tumour ROI TACs extracted from single-tracer noise-free MLEM were used as references (see the dashed lines in Fig. 12). When considering the results obtained from the single-tracer noisy MLEM, the tumour ROI TACs closely matched the reference TACs. However, in dual-tracer separation, the tumour ROI TACs obtained from the v-MTCM separation failed to align with the references accurately due to the noise in the images, the short time-delayed interval between two tracer injections, and the local minimum solution [17, 37]. While the introduction of a 3 mm FWHM Gaussian kernel on the separated images obtained from v-MTCM can reduce the noise level at the voxel level (see earlier Subsection "Separated image quality"), the performance of the separated tumour ROI TACs was notably worse compared to those obtained using v-MTCM. This is primarily because post-smoothing introduces bias on the separated images. The performance of the separated tumour ROI TACs using IS-F4D was even more compromised due to the non-unique solution of the optimization problem. While the pure data-driven CED method successfully reduced noise in the separated activity images at the voxel level, it leads to non-smooth separated tumour ROI TACs. The separated tumour ROI TACs obtained with the proposed deep network are closer to the reference TACs compared to those obtained by the other methods. In addition, the sum of the separated tumour ROI TACs with the proposed method (highlighted by a solid black line) aligns with the reference dual-tracer tumour ROI TAC (highlighted in a dashed black line), indicating that the proposed method effectively separates the dual-tracer signals while preserving data consistency.

**Fig. 13:**
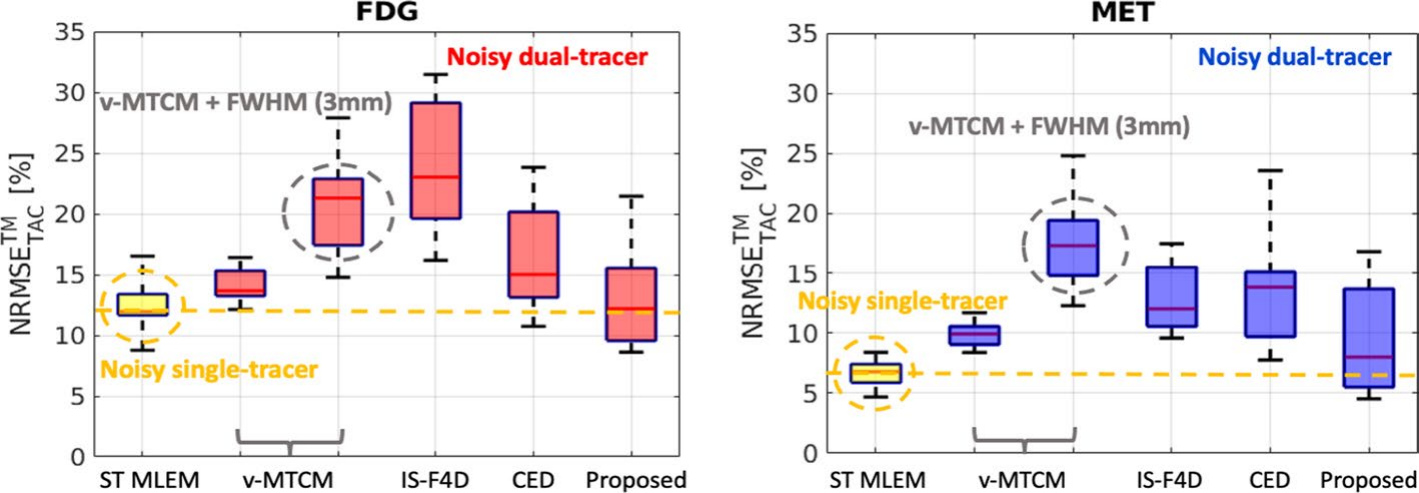
NRMSE of the tumour ROI TACs ($\text {NRMSE}_\text {TAC}^\text {TM}$) extracted from the separated single-tracer activity images (over 10 test examples, each with 20 different noise realisations) for FDG and MET, respectively. The yellow dashed lines indicate the median of the boxplots for the tumour ROI TACs extracted from the single-tracer noisy MLEM activity images

Figure 13 shows, on average, the single-tracer noisy MLEM achieved ${\sim }12\%$ and ${\sim }7\%$ $\text {NRMSE}_\text {TAC}^\text {TM}$ for FDG and MET, respectively. The $\text {NRMSE}_\text {TAC}^\text {TM}$ values of v-MTCM were slightly higher than those of the single-tracer noisy MLEM, resulting in ${\sim }14\%$ for FDG and ${\sim }10\%$ for MET. Higher $\text {NRMSE}_\text {TAC}^\text {TM}$ values were found with v-MTCM+FWHM (3 mm), IS-F4D and the CED when compared to the v-MTCM method. The proposed kinetic model-informed unrolled deep network achieved lower $\text {NRMSE}_\text {TAC}^\text {TM}$ in comparison to the two model-based methods and the CED. However, it is noteworthy that the single-tracer noisy MLEM still exhibited better performance with lower $\text {NRMSE}_\text {TAC}^\text {TM}$ (by comparing the median) for both FDG ($\sim$0.7%) and MET ($\sim$2%), compared to the proposed method.

## Discussion

This work proposes a kinetic model-informed DL-based method that incorporates a general kinetic model based on spectral analysis with the unrolled image-space version of the fully 4D method to separate dual-tracer [$^{18}$F]FDG+[$^{11}$C]MET activity images into single-tracer activity images without the need for any AIFs to be known or supplied to the method. Compared to the model-based methods (v-MTCM and IS-F4D), the proposed kinetic model-informed method can substantially reduce both the bias and SD of the separated activity images, as shown in Fig. 6. Compared to the pure data-driven CED method [37], which trains an over-parameterized neural network with a weak inductive prior for separation, the proposed method embeds a stronger inductive prior, i.e., the kinetic model, into the unrolled deep network for separation, resulting in even lower bias and SD with less training data. In addition, the bias level of the proposed method is comparable to the results obtained from single-tracer data using v-STCM in the separated activity image sequences for both FDG and MET.

To further evaluate the separation performance, the parametric maps $K_1$ and $K_i$ estimated from the separated single-tracer activity images using different methods was investigated. On average, v-MTCM and IS-F4D achieved over $100\%$ voxel-level NRMSE on the separated $K_1$ and $K_i$ images. The v-MTCM+FWHM (3 mm) method can reduce NRMSE but leads to over-smooth parametric images. Much lower NRMSE values were found for the DL-based methods (${<}30\%$ for both FDG and MET in $K_1$ and $K_i$), as shown in Fig. 9 and Fig. 11. Compared to the pure data-driven CED, the proposed method further reduced the NRMSE of the separated $K_1$ images by ${\sim }4\%$ for FDG and ${\sim }10\%$ for MET, (and by ${\sim }10\%$ for both FDG and MET in $K_i$), respectively.

ROI-level analysis was also performed by examining the separated single-tracer tumour ROI TACs to further assess the performance of the proposed method. On average, the v-MTCM method achieved lower $\text {NRMSE}_\text {TAC}^\text {TM}$ compared to those obtained by using v-MTCM+FWHM (3 mm), IS-F4D and the CED. The proposed method reduced the $\text {NRMSE}_\text {TAC}^\text {TM}$ by ${\sim }2\%$ for FDG, and ${\sim }4\%$ for MET, compared to the v-MTCM method. However, the $\text {NRMSE}_\text {TAC}^\text {TM}$ of the proposed method failed to get to a similar level to that of the single-tracer noisy MLEM, indicating that the separation capability of the proposed approach for tumour ROI TACs is still in need of further improvement.

Although the proposed method achieved a better separation performance compared to the model-based methods and the pure data-driven CED for mPET image separation at both the voxel and ROI levels, five main limitations should still be borne in mind. (1) The impacts of (i) the relative and absolute injected dose between the two tracers, (ii) the order of tracer injection, and (iii) the scanning protocol, were not investigated in this present study. (2) The current simulation study only focuses on separation for 2D PET (with a simple simulation of data acquisition) while conventional PET imaging is usually conducted in 3D. The proposed model would need to be investigated and validated rigorously on datasets obtained from physical phantoms with real data acquisition or synthetic data generated from real patient data to assess feasibility for practical application. (3) Even when 3D PET imaging is considered, low-count levels encountered in short time frames can lead to extremely noisy MLEM reconstructions, which makes the mPET image separation task even more challenging. With new techniques for enhancing the quality of reconstructed PET images and the arrival of new scanners, e.g., total-body PET, the higher-quality image data that includes different kinetics in various organs (since all organs would be in the field of view) could provide distinct features to enhance mPET image separation. (4) Feng’s input function model was employed merely to simulate the AIFs for each tracer in our current study. However, many AIFs cannot be fitted to this model, and alternative models have been proposed in [55–57]. The impact of different types of AIF would need to be further investigated. (5) The current study exclusively focuses on the dual-tracer separation of brain images using the tracer combination of [$^{18}$F]FDG and [$^{11}$C]MET. Additional investigations are required to explore the application of the proposed framework to other mPET separation tasks, such as the separation of mPET myocardial images using different tracer combinations [38].

It is worth noting that combining reconstruction with separation has been shown to enhance the separation performance in mPET imaging, as discussed in [17, 40]. While, in the present study, the separation process was conducted in the image domain, extending the proposed method to a direct mPET reconstruction-separation framework is achievable. This can be accomplished by integrating the system matrix into the proposed unrolled deep network, building upon the regularised version of the original fully 4D PET reconstruction algorithm [42].

The proposed method encounters a notable challenge in generalisation, particularly when applied to different tracer combinations or unseen images that are outside the training distribution. Another direction of potential future work could focus on performing fine-tuning on the pre-trained model using the unseen dataset in a self-supervised manner [58] to enhance its generalisation capability.

## Conclusion

We have developed a kinetic model-informed unrolled DL-based method for mPET image separation. Distinct from the pure data-driven deep learning methods, the proposed method improves the inductive prior by embedding the general kinetic model into an unrolled neural network based on a regularised image-space version of a fully 4D PET reconstruction algorithm. The proposed method has been developed to improve the quality of the separated single-tracer activity images and validated using simulated brain imaging data. In comparison to the pure data-driven CED, our proposed method can further reduce the bias and SD while requiring less data for training. It also achieved separation performance comparable to that obtained using single-tracer data, highlighting its potential for mPET image separation.

## Figures and Tables

**Algorithm 1:**
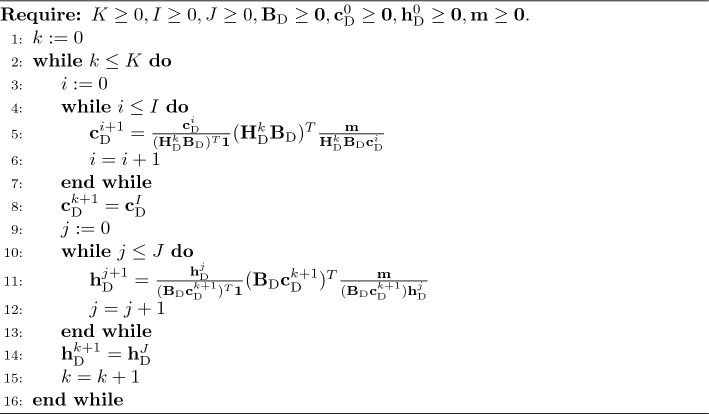
Image-space version of the fully 4D algorithm for dual-tracer PET image separation (IS-F4D) [20, 42]

## Data Availability

The datasets generated during and/or analyzed during the current study are available from the corresponding author on reasonable request.

## Abbreviations

mPET: Multiplexed positron emission tomography
TAC: Time-activity curve
AIF: Arterial input function
ROI: Region of interest
DL: Deep learning
CED: Convolutional encoder–decoder
CNN: Convolutional neural network
v-MTMC: Voxel-wise multi-tracer compartment modeling method
v-STMC: Voxel-wise single-tracer compartment modeling method
IS-F4D: Image-space dual-tracer version of the fully 4D PET image reconstruction algorithm
BN: Batch normalization
WM: White matter
GM: Grey matter
TM: Tumour
NF: Noise-free
ST: Single-tracer
DT: Dual-tracer
MSE: Mean square error
VWLS: Voxel-wise weighted least squares
FWHM: Full-width half maximum
NRMSE: Normalised root mean square error
SD: Standard deviation

## Footnotes

1 https://brainweb.bic.mni.mcgill.ca/brainweb/.

2 While we do model decay, we do not seek to include decay correction on the separated images in this present work.
